# Spinal Non-Hodgkin Lymphoma Mimicking Epidural Hematoma

**DOI:** 10.5334/jbsr.2928

**Published:** 2022-12-14

**Authors:** Lorenz Vangeel, Joris Bleyen, Laurens De Cocker

**Affiliations:** 1 AZ Maria Middelares Gent, BE

**Keywords:** spine, epidural, lymphoma, hematoma, MRI

## Abstract

A patient, recently diagnosed with non-Hodgkin lymphoma, presented with acute tetraplegia after surgical cervical lymph node biopsy. MRI of the cervical spine demonstrated an epidural space-occupying lesion with compressive myelopathy. While epidural hematoma was the tentative diagnosis, intra-operatively non-Hodgkin lymphoma was found. Several factors may have accounted for the inaccurate interpretation of the MRI: the acute clinical presentation appearing shortly after surgery, the non-specific signal intensities of (hyper-) acute hematomas, the lack of contrast-enhanced images, and the absence of the FDG-avid spinal mass in the PET/CT-report. Without radiological features of invasiveness and contrast-enhanced images, careful interpretation is mandatory for space-occupying epidural lesions.

**Teaching Point:**

Caution is needed when interpreting an epidural space-occupying lesion in the absence of contrast-enhanced images.

## Introduction

Non-Hodgkin lymphoma is the most common hematological malignancy worldwide, comprising 3% of all cancer diagnosis [1]. Lymphoma arises mostly in the lymphatic system, however extranodal involvement can occur throughout the body. Central nervous system involvement in the spinal canal is an uncommon extranodal involvement, nevertheless with possible devastating secondary neurological deficits.

## Case History

A 60-year-old woman with general malaise and dyspnea presented at the emergency department. Clinical history and physical examination were unremarkable. Computed tomography (CT) scan of the thorax and abdomen revealed extensive mediastinal and retroperitoneal mass lesions. Positron emission tomography – CT (PET-CT) scan showed fluorodeoxyglucose (FDG) avid masses throughout the body consistent with lymphoma. Excisional cervical lymph node biopsy was performed under general anaesthesia, and pathological analysis confirmed aggressive B-cell non-Hodgkin lymphoma.

**Figure 1:**
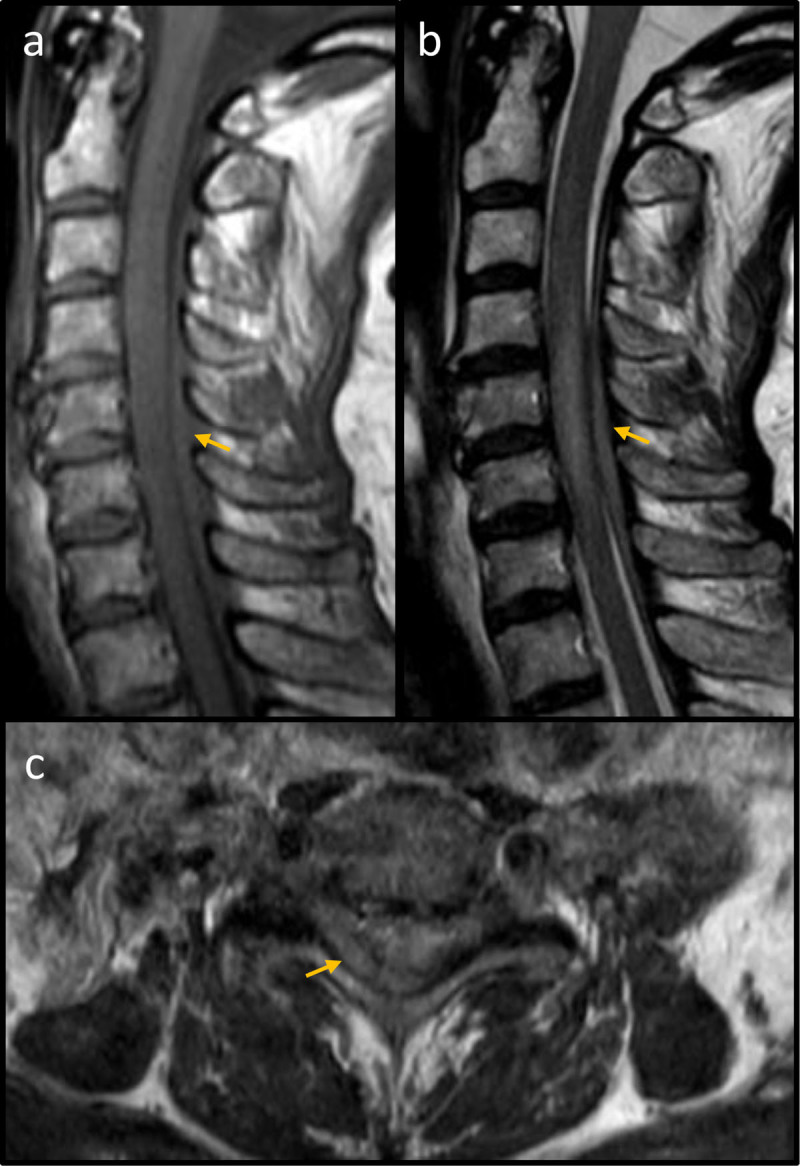
MRI of the cervical spine. **(a)** Sagittal T1 spin echo demonstrating an iso-intense space-occupying lesion (yellow arrow) restricted to the posterior epidural space. **(b)** Sagittal T2 spin echo showing a slightly hyper-intense mass (yellow arrow) in the posterior epidural space with corresponding myelopathy. **(c)** Axial T2 spin echo at vertebral level C6 showing a predominantly right-sided mass in the posterior epidural space (yellow arrow).

Unfortunately, the patient developed acute tetraplegia the first post-operative day. Magnetic resonance imaging (MRI) of the cervical spine revealed a space-occupying lesion in the posterior epidural space (Figure 1) with fusiform extension from C4 to C7. The spinal cord was found to be oedematous at the corresponding levels consistent with compressive myelopathy. An acute epidural hematoma was suspected, but during decompressive laminectomy a solid mass lesion was found and confirmed as lymphoma on histological examination.

**Figure 2:**
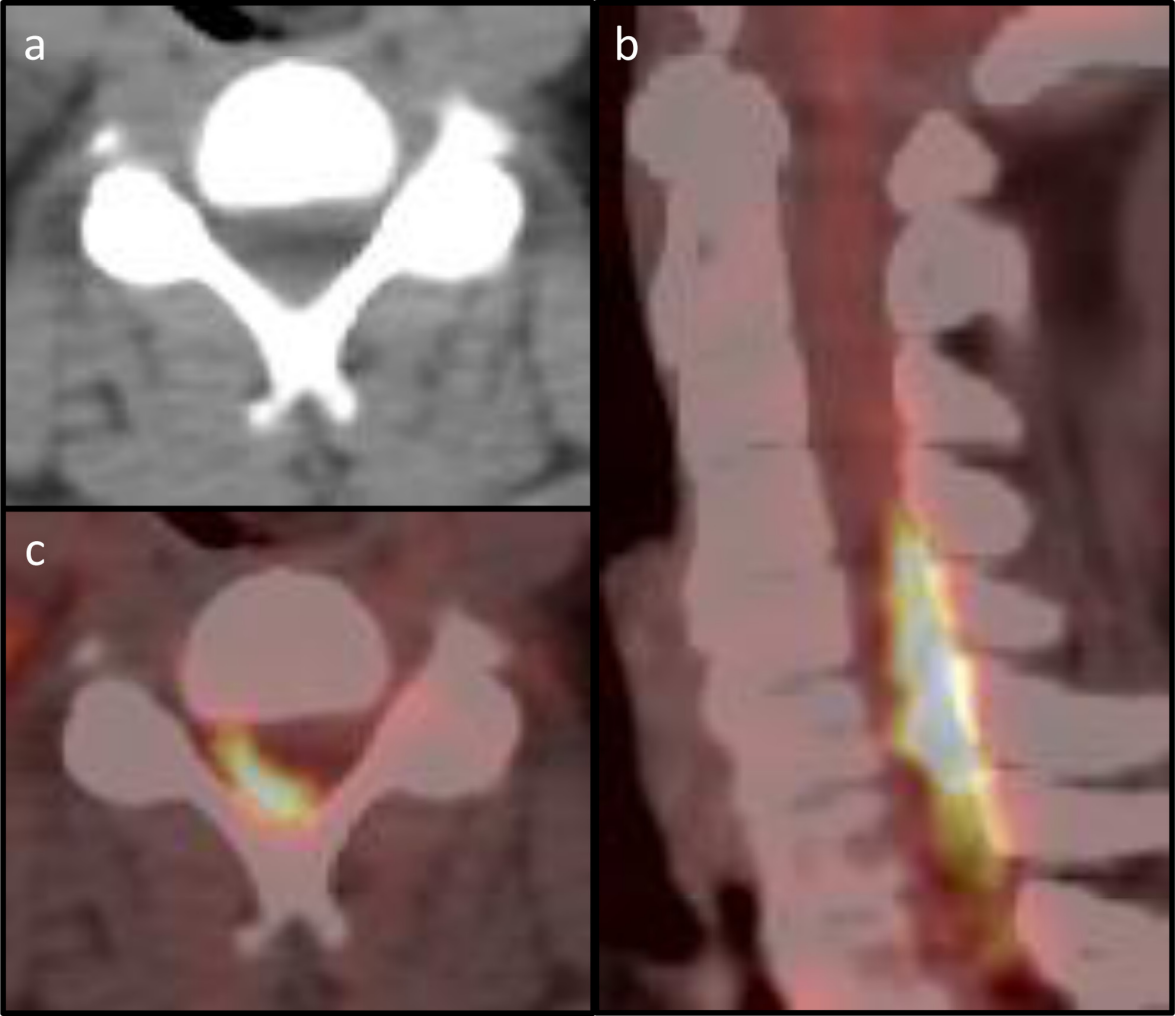
PET-CT of the cervical spine. **(a)** Axial CT at vertebral level C4 with barely visible posterior epidural mass. **(b)** Axial PET-CT of vertebral level C4 with notable FDG-avid disease in the spinal canal. **(c)** Sagittal PET-CT with FDG-avid disease posteriorly within the spinal canal.

In retrospect, the FDG-PET-CT scan already showed FDG-avid disease in the cervical spinal canal consistent with intraspinal extradural lymphoma, which was inconspicuous on the cross-sectional anatomical information provided by the CT part of the examination (Figure 2).

## Comment

Secondary central nervous system (CNS) lymphoma indicates CNS spread of (typically non-Hodgkin) lymphoma originating elsewhere. Secondary spinal lymphomas are more frequent than primary lymphomas [2]. Intraspinal location of non-Hodgkin lymphoma usually occurs in the extradural space, where it can be diagnosed in 0.1%–6.5% of patients with non-Hodgkin lymphomas [2,3].

Several clinical, radiological and technical features of the epidural lymphoma in this patient may have contributed to spinal lesion being mistaken for an hematoma initially.

First, the (hyper-)acute symptomatology of spinal cord compression might rather have been suggestive of a rapidly growing expansile process, such as a hematoma. It is unclear to which degree peri-operative manipulation of the neck (for an excisional cervical lymph node biopsy) could have triggered the acuteness of this clinical presentation.

Second, the radiological features of the lymphoma were erroneously interpreted as a hematoma. As for hematomas, the lymphoma in this patient strictly respected the anatomical boundaries of the epidural space, with normal (bony) posterior elements and no extension in either the intradural or interspinous spaces. In addition, the signal characteristics of an epidural lymphoma may resemble those observed in an hyperacute or acute hematoma on both T1- and T2 turbo spin echo-sequences, before the spontaneously bright T1-signal of methaemoglobin emerges in the subacute stage [4].

Third, contrast enhanced MRI was not performed because the clinical history was inappropriately taken into consideration. Homogeneous contrast enhancement as expected in lymphoma would have made this a more straightforward diagnosis, in particular in the clinical setting of systemic lymphoma [4].

Last, despite avid FDG-uptake, involvement of the spinal canal was overlooked on PET-CT performed days before the onset of paraplegia.

In conclusion, lymphoma should be considered in case of an epidural space-occupying lesion presenting with spinal cord compression. Since the majority of spinal lymphomas represent secondary disease, close correlation with clinical history is essential. Lack of invasive imaging features should not withhold the radiologist from gadolinium administration in this clinical setting.
